# Precise and insufficient scientific endeavour: COVID-19 pandemic experience from Bosnia and Herzegovina

**DOI:** 10.7189/jogh.13.03055

**Published:** 2023-11-03

**Authors:** Adnan Fojnica, Semir Vranic

**Affiliations:** 1 Molecular Biology and Biochemistry, Gottfried Schatz Research Center, Medical University of Graz, Graz, Austria; 2 College of Medicine, QU Health, Qatar University, Doha, Qatar

## PRECISE AND INSUFFICIENT SCIENTIFIC ENDEAVOUR

The coronavirus 2019 (COVID-19) pandemic has exacted a heavy burden for healthcare systems and medical professionals globally. Countries with a substantial portion of unvaccinated populations face difficulties in defending against the severe acute respiratory virus 2 (SARS-CoV-2) [1,2]. Mass rejection of COVID-19 vaccines in many countries and lack of collective awareness for vaccination have raised global concerns for current and could lead to even more pressing threats for future pandemics [2,3].

Above all, a rising trend in vaccine rejection seen over the years, fast development of COVID-19 vaccines, and strong opposition by the anti-vaccination movement led to the lack of COVID-19 vaccine acceptance on a global scale, including developing countries like Bosnia and Herzegovina (B&H).

In January 2021, B&H had the fourth-highest mortality rate (123 deaths reported per 100 000) from COVID-19 globally [3]. Despite this, the anti-vaccination movement remained adamant and did not divert even slightly from the general trend of vaccine rejection. The strong impact of the movement in B&H was first recognised during the 2014 measles outbreak [4]. Of 3804 measles cases identified, 70% were in unvaccinated patients [4]. The reasons for such behavior were not well understood. Initially, the trend was perceived as completely random and spontaneous phenomenon, lacking any underlying agenda. However, with the COVID-19 pandemic and our published work, it became evident that the problem is rooted deep in our society on several levels [3].

In early 2021, before vaccines were made available to the Bosnian population, we surveyed 10 471 individuals from B&H [3] and developed a predictive model assessing public response to COVID-19 vaccination. Surprisingly, only ~ 25% of people reported willingness to get vaccinated, providing varying rationale for their decision [3]. More than 50% of the population expressed a lack of trust in the medical profession and pharmaceutical companies as their reason for rejecting COVID-19 vaccines [3]. Around 20% believe that SARS-CoV-2 does not exist, while an almost identical percentage consider COVID-19 not dangerous for human health [3]. Healthcare professionals showed a comparable trend of low vaccine acceptance; a similar trend had been previously observed in lieu of measles vaccines [3,5,6].

**Figure 1:**
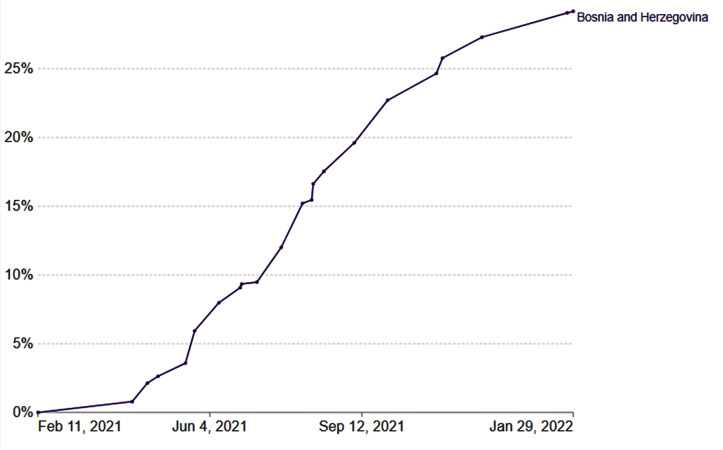
Data indicate the share of people who received at least one dose of the COVID-19 vaccine. Obtained from Our World in Data [7].

Today, after 16 346 reported deaths and more than 400 000 confirmed cases of COVID-19 in B&H, only ~ 25% of people have been fully vaccinated (**Figure 1**), aligning with our predictions [7,8]. Having a model that predicts outcomes with 98% precision would typically be regarded as a highly successful endeavour. However, reality leaves little room for elation, as unvaccinated individuals are vulnerable hosts for the widespread disease and as SARS-CoV-2 constantly mutates. Despite the mass attention our study received in local media and social networks, little progress has been made in addressing these issues in the community. This scenario exemplifies a textbook case of how problems, although recognised and acknowledged, often become challenging to resolve effectively.

A significant disparity exists between conducted research and the information accessible to the general population in B&H. People lack awareness of scientific progress, and scientific literature is reported to be read by a small percentage of the population, mainly healthcare professionals [3].

The proposed solution needs to include scientists stepping out of the laboratories to bridge the gap between scientific knowledge and public understanding of vaccination and the COVID-19 pandemic. Additionally, to enhance public understanding of the health advantages associated with vaccination and the critical role it has played in eradicating numerous diseases in the past, it is crucial to effectively engage individuals through primary information channels, such as educational initiatives and media platforms, as they have been the primary sources of information during the pandemic [3]. Scientifically verified and reliable information needs to be delivered to the population, while fake news must be actively filtered out. As healthcare professionals directly communicate with patients and shape their perspective toward vaccination, a proactive approach is required to facilitate scientific panels and conferences designed for healthcare workers and physicians to raise societal awareness [9]. A comprehensive and multidirectional approach is key to effectively mitigating vaccine hesitancy among healthcare professionals. This approach includes the implementation of targeted informational campaigns structured to reinforce evidence-based understanding of vaccine-related issues. Respected scientists advocating vaccination publicly could serve as exemplars among healthcare professionals with low acceptance rates. Further, the strategic deployment of testimonials, exemplifying the positive outcomes of vaccination, can significantly impact the attitudes of healthcare professionals.

Science relies heavily on collective efforts and collaborations on multiple levels, from the individual to the politicians. In our case, we found obstacles on every level [3,5]. The proposed solutions must be implemented systematically, as reports indicate problems deep down in education, healthcare systems, and governmental policies [3,5,6].
